# Screening for thyroid disorders in asymptomatic adults from Brazilian populations

**DOI:** 10.1590/S1516-31802002000500005

**Published:** 2002-09-02

**Authors:** Isabela Benseñor

**Keywords:** Subclinical, Hypothyroidism, Symptoms, Thyroid, Disorders, Cardiovascular disease, Hipotireoidismo, Subclínico, Sintomas, Doenças da tireóide, Doenças cardiovasculares

## Abstract

Advances in thyroid disorder diagnosis have created new thyroid disorder categories such as sub-clinical hyperthyroidism and subclinical hypothyroidism. In the 1980s, immunometric assaying for thyroid stimulating hormone (TSH) emerged and became defined as the most cost-effective test in thyroid disorder screening. The second step in the screening of thyroid disorders is to determine free thyroxine (FT4), and cost-effective methods for its detection are now available. Using TSH and FT4, it is possible to determine four situations: clinical hyperthyroidism, clinical hypothyroidism, subclinical hyperthyroidism and subclinical hypothyroidism. Subclinical hypothyroidism can be a strong indicator of risk for atherosclerosis and myocardial infarction in elderly women. Cardiovascular mortality among Brazilian women is one of the highest in the Western world. The best-known risk factors for cardiovascular diseases are high blood pressure, smoking, diabetes, and hypercholesterolemia. Although these are recognized as primary risk factors, there are other risk factors that could be identified as primordial risk factors. This may be the case for subclinical hypothyroidism. Early detection of thyroid disorders in women over fifty could be a highly cost-effective option in the prevention of cardiovascular disorders among Brazilian women.

## RESUMO

O avanço no diagnóstico das doenças da tireóide criou uma nova categoria de doenças representada pelo hipertireoidismo e hipotireoidismo subclínicos. Na década de 80, surgiram os métodos imunométricos para dosagem da tireotropina (TSH) considerados os mais custo-efetivos para o rastreamento de doenças da tireóide. O segundo passo no rastreamento das doenças da tireóide é a determinação dos níveis séricos de tiroxina, e vários métodos custo-efetivos estão disponíveis no mercado. Usando-se o TSH e a tiroxina é possível diagnosticar quatro situações clínicas: hipotireoidismo e hipertireoidismo e hipotireoidismo subclínico e hipertireoidismo subclínico.

O hipotireoidismo subclínico pode ser um fator de risco muito importante para doença cardiovascular em mulheres idosas. A mortalidade cardiovascular nas mulheres brasileiras é uma das mais elevadas do mundo oci-dental. Os fatores de risco para doença cardiovascular mais conhecidos são tabagismo, hipercolesterolemia, hipertensão arterial e diabetes mellitus. Esses fatores, tidos como primários, podem ser conseqüência de fatores de risco primordiais como pode ser o caso do hipotireoidismo subclínico. A detecção precoce de doenças da tireóide em mulheres com mais de 50 anos pode ser uma opção custo-efetiva na prevenção das doenças cardiovasculares nas mulheres brasileiras.

## INTRODUCTION

Advances in thyroid disorder diagnosis have created a new category of thyroid disorders such as subclinical hyperthyroidism and subclinical hypothyroidism. In these two conditions, serum levels of free thyroxine and triiodothyronine are normal, but serum thyrotropin levels are mildly decreased in sub-clinical hyperthyroidism and mildly elevated in subclinical hypothyroidism. This fact has a particular relevance in Brazil because of the higher cardiovascular mortality in our women^1^ that could be associated with possible subclinical hypothyroidism.

The US Preventive Services Task Force does not recommend screening for thyroid disorders.^2^ Neither does the British Royal College of Physicians, among adults.^3^ How-ever, there is no consensus about this and a lot of conflicting recommendations exist. The American Thyroid Association recommends the screening of women and men aged 35 years or over.^4^ The American Association of Clinical Endocrinologists recommends screening among older women.^5^ The American College of Pathologists recommends screening for women aged over 50 years who seek medical care and for all geriatric patients upon hospital admission.^6^ The American Academy of Family Physicians recommends screening for patients aged over 60 years, independent of gender.^7^ The American College of Physicians recommends screening for women aged over 50 years with unspecific complaints.^8,9^ In particular, the American College of Obstetrics and Gynecology recommends screening for high risk groups like women with a family history of thyroid or autoimmune diseases, from the age of 20 years onwards, so as to prevent fetal disorders.^10^

Discussion of this issue has been considered to be so important that the New England Journal of Medicine recently published two reviews analyzing evidence for the treatment of subclinical hypothyroidism and subclinical hyperthyroidism and their possible complications.^11,12^

In this review paper, we will discuss how to adapt guidelines for the screening of thyroid disorders to Brazilian populations, including analyses based on the local prevalence of thyroid and cardiovascular diseases.

### Prevalence of hyper and hypothyroidism

One of the most important data sets regarding thyroid diseases has come from the Whickham Survey, undertaken in Britain in 1975.^13^ The Whickham Survey observed that thyroid stimulating hormone (TSH) did not vary with age among males but increased markedly among females after the age of 45 years. The rise in TSH levels with age among females was virtually abolished when persons with thyroid antibodies were excluded from the sample.

Twenty years later, a follow-up of the Whickham Survey evaluated the incidence and natural history of thyroid disease in this cohort. Of the 1,877 survivors, 96% participated in the follow-up and 91% were tested for clinical, biochemical and immunological evidence of thyroid dysfunction.^14^ The risk of having developed hypothyroidism by the time of the follow-up, among people with raised TSH levels alone, was 8 (3 – 20) for women and 44 (19 – 104) for men, expressed as the odds ratio (OR) with 95% confidence interval (95% CI). The risk of having developed hypothyroidism by the time of the follow-up, among people with positive thyroid antibodies alone, was 8 (5 – 15) for women and 25 (10 – 63) for men. The risk among people with both risk factors (raised serum TSH levels and positive thyroid antibodies) was 38 (22 – 65) for women and 173 (81 – 370) for men. Neither a positive family history of thyroid disease nor the parity of women at the time of the first survey was associated with increased risk of developing hypothyroidism.^14^

In an unselected population of elderly people (the 1985 Framingham cohort), the prevalence of thyroid deficiency, as shown up by clearly elevated TSH levels that exceeded 10 mU/ml, was 4.4%. Women (5.9%) had thyroid deficiency more often than men (2.3%). Of those with clearly elevated serum TSH levels, only 39% had low serum thyroxine (T4) levels. The conclusion was that an elevated serum TSH level was a sensitive marker for thyroid deficiency in the elderly and was often the only way to detect it.^15^

### Methodology for screening

Until the first half of the 1980s, the laboratory diagnosis of thyroid dysfunction was obtained through radioimmunoassay for TSH. This did not detect decreased values of TSH, and it is not a good test for hyperthyroidism diagnosis. In the latter part of the 1980s, immunometric assaying for TSH emerged and became defined as the most cost-effective test in thyroid disease screening.^16^ Second-generation immunoassays can detect TSH values of 0.1 mIU/l and third-generation assays are able to detect TSH values of 0.01 mIU/l. Below such levels, TSH is undetectable.

### Rationale for using TSH as the primary thyroid function test

#### Thyroid function

Thyroid hormone production by the thyroid gland is stimulated by TSH, which is synthesized and secreted from the anterior pituitary. In patients with an intact hypothalamic-pituitary-thyroid axis, a negative feedback regulatory mechanism controls thyroid gland metabolism. The pituitary serves as a biosensor of thyroid hormone levels and regulates TSH levels according to the feedback of free thyroxine (FT4) and free triiodothyronine (FT3) levels. Decreases in thyroid hormone production stimulate more TSH secretion. The control system may have a relatively slow response time. During periods of non-equilibrium (beginning of hypothyroidism and during hyperthyroidism treatment), discordance may occur between the plasma thyroid hormone concentrations and the levels of TSH.

There are three main reasons why TSH is a good test for detecting thyroid disease. There is an inverse log-linear relationship between the concentrations of TSH and FT4. Small linear decreases in FT4 concentrations are associated with exponential decreases in TSH concentrations. Almost all the cases of hypothyroidism and hyperthyroidism encountered in general medical practice are caused by primary disease of the thyroid gland. A further reason is that immunometric assays for TSH are very sensitive and specific tests, whose sensitivity and specificity are higher than 99%. ^6,16^

The second step in the screening of thyroid disorders is to determine FT4. Thyroglobulin (thyroxine-binding globulin) levels may vary in a great number of disorders, but do not affect FT4. The use of FT4 now presents good cost-effectiveness in comparison with classical methods. Through the use of TSH and FT4, four situations can be defined: clinical hyperthyroidism, clinical hypothyroidism, subclinical hyperthyroidism and subclinical hypothyroidism. ^9^

There is no consensus regarding the definitions of subclinical hyperthyroidism and hypothyroidism. Some guidelines define subclinical hypothyroidism as a situation in which the patient is asymptomatic, and others accept this definition when the patient has a few symptoms, but without any definition of what symptoms have to be considered. ^4–6^ The same problem occurs in relation to subclinical hyperthyroidism. Would carefully obtaining the history and making a physical examination followed by some selected tests constitute better screening for detecting thyroid dysfunction than the screening of the whole population?

Two papers have addressed this question. In England in 1988, screening performed on 2,000 consecutive patients seen at a primary care center detected 19 cases of hypothyroidism or hyperthyroidism. On the basis of their own clinical assessments, clinicians ordered thyroid function tests on 35 of the 2,000 patients, none of whom were found to have thyroid disease.^17^

In another study, the screening of 1,152 Swedish women detected three cases of hypothyroidism and two cases of hyperthyroidism. On the basis of the history and physical examination, the clinicians tested 286 women but failed to diagnose the condition in any of the three women with hypothyroidism and in one of the two who had hyperthyroidism.^18^

These two papers may demonstrate that the history and physical examination are not sensitive or specific for thyroid disease diagnosis. However, these data can be analyzed from a different perspective. Clinicians cannot consider that unspecific symptoms such as fatigue malaise, small gains or losses in weight, anxiety or depressive symptoms are signs of hyperthyroidism or hypothyroidism. This fact takes on greater importance when special risk groups such as women aged over 50 are considered.

#### Why should subclinical hyperthyroidism be treated?

The prevalence of subclinical hyperthyroidism is about 1% (range 0.4% - 1.7%) in men aged over 60 years, and 1.5% (0.8% - 2.5%) in women aged over 60 years.^19^ The main potential benefit of treating subclinical hyperthyroidism is prevention of atrial fibrillation and osteoporosis.^9^ No randomized trials have been performed in relation to early treatment for subclinical hyperthyroidism, nor have studies been made to evaluate functional status or quality of life among patients with subclinical hyperthyroidism identified by screening.

Persons over 60 years old with diminished TSH levels are at increased risk for atrial fibrillation. In the Framingham Cohort, 2,007 persons (814 men and 1,193 women) were studied who were 60 years of age or older and who did not have atrial fibrillation, in order to determine the frequency of this arrhythmia over a 10-year follow-up period. After multivariate adjustment, the relative risk of atrial fibrillation among elderly subjects with low serum thyrotropin concentrations, as compared with those with normal concentrations, was 3.1 (95% CI of 1.7 – 5.5). It was concluded that, among people aged 60 years or older, a low serum thyrotropin concentration is associated with a threefold higher risk for the development of atrial fibrillation over the subsequent decade.^20^ Assuming that treatment would reduce the risk for atrial fibrillation to that of the general population, the number needed to treat (NNT) to prevent one case of atrial fibrillation over 10 years would be 4.2.

A recent study has shown increased mortality from all causes, and in particular mortality due to circulatory and cardiovascular diseases, among individuals aged 60 years and older who had subclinical hyperthyroidism diagnosed but were not receiving any treatment. The increases in mortality from all causes in years 2 to 5 were greater among patients with low serum thyrotropin than for the remainder of the cohort (the hazard ratios for years 2, 3, 4 and 5 were 2.1, 2.2, 1.8, and 1.8, respectively). This reflected increased mortality from circulatory diseases (the hazard ratios for years 2, 3, 4 and 5 were 2.3, 2.6, 2.3, and 2.3) and cardiovascular diseases (the hazard ratios for years 2, 3, 4 and 5 were 3.3, 3.0, 2.3, and 2.2).^21^

Information about the risk of osteoporosis in subclinical hyperthyroidism is scant. Two cross-sectional studies have evaluated bone density in patients with subclinical hyperthyroidism associated with multinodular goiter. The patients in these studies had statistically and clinically significant decreases in bone mineral density at the femoral neck and at the radius, in comparison with age-matched controls.^22,23^

In a nested case-control study from within the Study of Osteoporotic Fractures cohort, women with low TSH levels had an increased risk for hip or vertebral fracture over a period of 4 years (OR of 2.5; 95 % CI of 1.1 – 8.0). In many of these women, however, the low serum TSH level was associated with a history of hyperthyroidism or use of L-thyroxine. The fracture risk among women with low serum TSH levels but no history of clinical thyroid disease had not previously been reported.^22^ However, up until that time, there had not been any good evidence that subclinical hyperthyroidism could be associated with an increased risk of osteoporosis, and this needed to be confirmed in further studies.^23,24^

#### Why should subclinical hypothyroidism be treated?

Subclinical hypothyroidism is the most common condition found by screening using thyroid function tests. Five to 10% of adult women have an elevated TSH level.^13,14^ The potential complications of subclinical hypothyroidism are elevations in serous levels of total cholesterol and its fractions, and progression to overt hypothyroidism. Most patients found by screening to have subclinical hypothyroidism have at least one symptom that could be related to this diagnosis. These symptoms include muscle cramps, dry skin, intolerance to cold, constipation, poor energy levels, fatigue, and mental slowness.^25,26^ Data from randomized trials on the effects of Lthyroxine treatment in subclinical hypothyroidism are to be found in Table 1.

**Table 1:** Randomized trials for the treatment of subclinical hypothyroidism

	Cooper et al. (1984)^27^	Nyström et al. (1988)^28^	Jaeschke et al. (1996)^29^
Design	Randomized, double-blind, placebo-controlled trial with 1-year follow-up	Randomized, double-blind, crossover trial with 1 -year follow-up (6 months for placebo and the same for hormone)	Randomized, double-blind, placebo controlled trial with 11 months of follow-up
Country (city)	USA (Boston, MA)	Sweden (Gothenburg)	Canada (Hamilton, Ontario)
Selection of study sample	Endocrine referral clinic at academic center	Population-based study with women for screening selected from the general population	Outpatient clinic in a community hospital
Description of study sample	Patients with subclinical hypothyroidism after treatment with iodine-131, 20 years earlier	Women aged over 50 years with subclinical hypothyroidism diagnosed in a screening program	Patients aged over 55 years with subclinical hypothyroidism and symptoms consistent with hypothyroidism
Final sample (n)	41	20	37
Final size (n)	33	17	32
Women (%)	97	100	76
Mean age (years)	55	58	68
TSH reference range, mlU/l	< 0.5-3.5	0-4.0	< 0.5-5.5
Mean TSH level, mlU/l	10.9	7.7	9.9
Total cholesterol level, mmol/l	6.3	6.8	6.1
Symptoms per patient n/n	2.25	1.6	NA
**Results**			
Placebo group (n)	16	8	16
Patients who improved, (%)	19	NA	50
Patients who worsened, (%)	24	11	31
Treatment group (n)	17	9	16
Patients who improved (%)	47	25	42
Patients who worsened (%)	24	11	31
Number needed to treat*	3.6	4.5	Impossible to calculate
Lipid levels, total cholesterol, HDL, LDL, triglycerides	No difference between groups	No difference between groups	No difference between groups

*NA = not available; NS = not stated; Number needed to treat = number of patients that have to be treated to obtain one patient with an improvement in the condition. In the first study, for every 3.6 patients treated by Cooper et al., one improved. Adapted from American College of Physicians.^9^*

There are a lot of problems in the three trials presented in Table 1. The numbers of individuals sampled are very small, which makes analyses difficult. The patients sampled in the study by Cooper et al. were patients with hyperthyroidism treated using iodine-131 who became hypothyroid (subclinical).^27^ This is not an ideal sample for studying the treatment of subclinical hypothyroidism. In the second study, women were selected by screening but it was a double-blind study in which the doses of thyroxine were fixed (0.15 mg daily). Thus, many women persisted with symptoms, but in reality, they were not adequately treated since the physicians could not correctly adjust the TSH levels.^28^ In the third study, in almost half of the patients, the TSH values reverted to normal without treatment (the “regression to mean” phenomenon). The same occurred in the first and the second trials.^29^ Consequently, TSH levels will have to be confirmed in subsequent measurements in future trials before patients can be included in studies.

We now have the preliminary results from two small placebo-controlled trials. In the first study, on women whose thyrotropin levels were mildly elevated, thyroxine therapy had no effect on symptoms.^30^ However, the second trial showed positive results for thyroxine in the treatment of symptoms. The TSH levels in these women were higher than the levels in the first study.^31^

Treatment of subclinical hypothyroidism is recommended because of cholesterol levels. However, in these three studies in Table 1, the cholesterol levels did not change with treatment. In 1996, Danese et al., using a computerized model to calculate the cost-effectiveness of screening 35-year-old patients with a serum TSH assay every 5 years, found a value of $ 9,223 for quality-adjusted life years (QALYs) for women and $ 22,595 for men. In this analysis, Danese et al. assumed that among patients with a total cholesterol level greater than 6.2 mmol/l, treatment with Lthyroxine reduces the total cholesterol levels by 17%. They therefore concluded that it was more cost-effective to assay TSH levels and treat patients with L-thyroxine than to give drugs for lowering cholesterol levels in these people. However, the improvement in hyper-cholesterolemia after treatment with L-thyroxine was overestimated in this study.^32^ In a recent meta-analysis of 13 studies on patients with subclinical hypothyroidism, the pooled mean reduction in total cholesterol after Lthyroxine treatment was 0.4 mmol/L, which was associated with a decrease in cardiovascular risk of 8%. In this meta-analysis, most of the patients were not selected by screening, which may have meant that they were easier to treat than patients selected by screening.^33^

Cardiovascular disease in hypothyroid patients has been causally associated with hypercholesterolemia. However, recent studies suggest that there is a causal relation between hypothyroidism and cardiovascular disease, independent of the hypercholesterolemia. In a population-based cohort of 1,149 women (the Rotterdam Study), subclinical hypothyroidism was present in 10.8% of the sample and was also associated with a greater age-adjusted prevalence of aortic atherosclerosis (OR of 1.7; 95% CI of 1.1 – 2.6), and myocardial infarction (OR of 3.1; 95% CI of 1.5 – 6.3). The study concluded that subclinical hypothyroidism was a strong indicator of risk for atherosclerosis and myocardial infarction in elderly women.^34^ However, this was just a cross-sectional study and follow-up studies will be necessary to confirm these data.

A survey on the effects of smoking (another cardiovascular risk factor) among women with various grades of hypothyroidism, compared with normal women, concluded that smoking increased the metabolic effect of hypothyroidism in a dose-dependent way. Women with subclinical hypothyroidism who smoked had higher TSH levels, and higher ratios of serum triiodothyronine to serum free thyroxine. The serum concentration of total cholesterol and low-density lipoprotein (LDL) cholesterol were also higher in these women with subclinical hypothyroidism who smoked, in comparison with women with subclinical hypothyroidism who did not smoke.^35^

The potential risks of treating subclinical hypothyroidism are the side-effects of L-thyroxine replacement, including nervousness, palpitations, atrial fibrillation, and exacerbation of angina *pectoris*.

Adverse effects were reported in two of the three randomized trials that addressed the treatment of subclinical hypothyroidism. Overtreatment with L-thyroxine is another possibility, because almost 50% of hypothyroid patients treated with L-thyroxine are unintentionally maintained on doses that are sufficient to cause TSH suppression.

#### What is the importance of screening for thyroid disorders in Brazil?

Cardiovascular mortality among Brazilian women is one of the highest in the Western world. The best-known risk factors for cardiovascular diseases identified by cohort studies are high blood pressure, smoking, diabetes, and hypercholesterolemia. Although these are recognized as primary risk factors, there are other risk factors that may be identified as primordial risk factors, because they appear before primary risk factors. These primordial risk factors are high body mass index (BMI), high fat intake (including saturated, polyun-saturated, and trans-fatty acids), and high alcohol intake.^36^

The identification of such primordial factors is now one of the most important topics in research issues because of their potential for prevention in clinical practice. The possible primordial risk factors are related to occupational risks,^37^ social inequalities,^38^ and genetic polymorphism.^39^ However, the associations that these disorders have with non-cardiovascular diseases, and their preclinical manifestations are mostly unexplored, despite the important preventive potential of new risk factor identification in clinical practice. This may be the case with subclinical hypothyroidism, which is characterized by raised TSH levels and normal FT4 levels.

Recent studies like the Rotterdam Study^34^ have shown that, in the preclinical phase of hypothyroidism, there is a positive association between subclinical hypothyroidism and atherosclerotic manifestations, independent of cholesterol levels. The relationship tends to be stronger among women with positive antiperoxidase autoantibodies.

To obtain a better understanding of the importance of screening for subclinical hypothyroidism, it is very important to analyze the new data on cardiovascular diseases for Brazilian women, the pathophysiology of thyroid hormones, and the epidemiology of thyroid disorders in Brazil.

### Prevalence of thyroid disorders in Brazil

The epidemiological studies performed in Brazil have been relatively restricted in scope. Nonetheless, the data suggest that the prevalence of thyroid disorders in Brazil has considerable importance. One study on 300 autopsy cases that did not have clinical thyroid disorders showed the presence of benign and malignant thyroid cancer in 6.6% of them.^40^ Another study on 10,000 thyroid biopsies showed the presence of non-neoplastic lesions in 86.8% of the sample, with the majority of the cases being various types of goiter, Graves’ disease, and thyroiditis.^41^ In 1994, the prevalence of antiperoxidase antibodies was analyzed among 402 patients with thyroid disorders and 30 healthy controls. The thyroid disorders in this sample were cases of autoimmune disorders such as Graves’ disease and Hashimoto's thyroiditis, cancer, and hormone-generated goiter from an area with a mild iodine deficiency. The prevalence of antiperoxidase antibodies in this sample was 89.9% among patients with autoimmune diseases and 4.8% among patients without autoimmune disorders. In relation to antimicrosomal antibodies, there was a 68.4% positivity among patients with autoimmune disorders and 6.4% among patients without autoimmune disorders.^42^ In 1995, a study was made of an association with iodine nutritional deficiency, among 550 patients in an area with a prevalence of hormone-generated goiter, in which 5% of the patients were found to have subclinical hypothyroidism.^43^

### Prevention of cardiovascular disease among Brazilian women

Among women, the frequency of cardiovascular events is greater after the menopause, and this is associated with the reduced levels of estrogens, as demonstrated in observational studies. Such cardiovascular events have a greater impact on general mortality than breast cancer does.^44^ However, recent data from a clinical trial among women with previous cardiovascular disease (Heart and Estrogen/Progestin Replacement Study),^45^ and the initial results from the Women's Health Initiative mega-trial,^46^ have provided increased evidence that hormone use among menopausal women compared to placebo can be associated with higher risks of cardiovascular disease in the first two years. Therefore, at this moment, the identification of another risk factor associated with cardiovascular disease, either mediated by cholesterol levels or not, as in subclinical hypothyroidism, would represent a new approach, possibly with lower costs and risks than for hormonal use.

Another proposed approach for men and women is the primary prevention of cardiovascular disease treating hypercholesterolemia, using drugs like vastatins or fibrates. There are some reservations to this approach and the most important of them is the reduced cost-effectiveness of this practice.^47^ An alternative approach would be the detection of subclinical hypothyroidism as a primor-dial risk factor for cardiovascular disease among Brazilian women.

In addition to the possible impact on the prevention of cardiovascular diseases, treatment of subclinical hypothyroidism can help in the treatment of mild cases of depression,^48^ or unspecific symptoms such as fatigue, dizziness, and other symptoms associated with hypothyroidism that can be less typical manifestations of the disorder.

#### What is recommended for Brazilians?

There is substantial evidence that hypothyroidism may be a common problem for women aged over 50 years throughout the world. There is some evidence that this may be a real problem in Brazil and perhaps the presence of thyroid disorders can help explain why cardiovascular mortality among Brazilian women is so high in comparison with other countries.^49^ Consequently, hypothyroidism may be a linking factor for the higher mortality levels from cardiovascular disease among Brazilian women, which are only comparable with those in Eastern Europe (the highest in the world).^1^

So, what is best for Brazilians? Firstly, most of the possible effects from the high prevalence of thyroid disorders would be concentrated among Brazilian women. Therefore, the high-risk group that needs to be screened will be women aged over 50 years. Secondly, until an original study about the real prevalence of subclinical hypothyroidism is produced in Brazil, physicians in clinical fields will need to be very sensitive towards the diagnosis of clinical and subclinical hypothyroidism, especially among women with unspecific symptoms such as fatigue, mild weight gain and depression. New research will need to clarify points like whether such symptoms can be explained by different disorders, or whether they are comorbidities with a common causal link, or whether they are real manifestations of clinical hypothyroidism.

#### What do we propose?

There is a need for early detection of thyroid disorders, especially subclinical hypothyroidism, which is the most prevalent one among women aged over fifty.

For these women or for other people with a lot of unspecific symptoms that do not fulfill the criteria for psychiatric disorders like depression or anxiety, screening for thyroid disorders could be a simple option. Such screening for thyroid disorders could be done using just the TSH and FT4 levels.

## References

[1] Lotufo PA (1998). Premature mortality from heart diseases in Brazil. A comparison with other countries. Arq Bras Cardiol.

[2] Preventive Services Task Force (1996). Guide to clinical preventive services.

[3] Vanderpump MP, Allquist JA, Franklyn JA (1996). Consensus statement for good practice and audit measures in the management of hypothyroidism and hyperthyroidism. BMJ.

[4] Ladenson PW, Singer PA, Ain KB (2000). American Thyroid Association guidelines for detection of thyroid dysfunction. Arch Intern Med.

[5] (1996). Clinical practice guidelines for the evaluation and treatment of hyperthyroidism and hypothyroidism.

[6] Glenn GC (1996). Practice parameter on laboratory panel testing for screening and case finding in asymptomatic adults. Laboratory Testing Task Force of the College of the American Pathologists. Arch Pathol Lab Med.

[7] (2000). Periodic Health Examination: summary of AAFP policy recommendations and age charts, revision 4.0.

[8] American College of Physicians (1998). Screening for thyroid diseases. Annals Intern Med.

[9] American College of Physicians (1998). Screening for thyroid diseases. Annals Intern Med.

[10] (1998). An update in obstetrics and gynecology: primary & preventive care.

[11] Cooper DS (2001). Subclinical hypothyroidism. N Engl J Med.

[12] Toft AD (2001). Subclinical hyperthyroidism. N Engl J Med.

[13] Tunbridge WMG, Evered DC, Hall R (1977). The spectrum of thyroid disease in a community: the Whickham Survey. Clin Endocrinol.

[14] Vanderpump MPJ, Tunbridge WMG, French JM (1995). The incidence of thyroid disorders in the community: a twenty-year follow-up of the Whickham Survey. Clin Endocrinol.

[15] Sawin CT, Castelli WP, Hershman JM, McNamara P, Bacharat P (1985). The aging thyroid. Arch Intern Med.

[16] Klee GG, Hay ID (1997). Biochemical test of thyroid function. Endocrinol and Metab Clinics of North America.

[17] Eggertsen R, Petersen K, Lundberg PA, Nyström E, Lindstedt G (1988). Screening for thyroid disease in a primary care unit with a thyroid stimulating hormone assay with a low detection limit. BMJ.

[18] Petersen K, Lindstedt G, Lundberg PA, Bengtsson C, Lapidus L, Nyström E (1991). Thyroid disease in middle-aged and elderly Swedish women: thyroid-related hormones, thyroid dysfunction and goiter in relation to age and smoking. J Intern Med.

[19] Parle JV, Franklyn JA, Cross KW, Jones SC, Sheppard MC (1991). Prevalence and follow-up of abnormal thyrotropin levels (TSH) concentrations in the elderly in the United Kingdom. Clin Endocrinol (Oxf).

[20] Sawin CT, Geller A, Wolf PA (1994). Low serum thyrotropin concentrations as a risk factor for atrial fibrillation in older persons. N Eng J Med.

[21] Parle JV, Maisonneuve P, Sheppard MC, Boyle P, Franklyn JA (2001). Prediction of all-cause and cardiovascular mortality in elderly people from one low serum thyrotropin result: a 10-year cohort study. Lancet.

[22] Füldes J, Tarján G, Szathmari M (1993). Bone mineral density in patients with endogenous subclinical hyperthyroidism: is the thyroid status a risk factor for osteoporosis?. Clin Endocrinol (Oxf).

[23] Mudde AH, Reijnders FJ, Kruseman AC (1992). Peripheral bone density in women with untreated multinodular goiter. Clin Endocrinol (Oxf).

[24] Bauer DC, Ettinger B, Newitt MC, Stone KL, Cummings SR (1995). Women with low serum TSH have an increased risk of hip and vertebral fractures: a prospective study. The Study of Osteoporotic Fractures Research Group. J Bone Miner Res.

[25] Tofi AD (1994). Thyroxine Therapy. N Engl J Med.

[26] Franklyn JA, Berteridge J, Dykin J (1992). Long-term thyroxine treatment and bone mineral density. Lancet.

[27] Cooper DS, Halpern R, Wood LC (1984). L-thyroxine therapy in subclinical hypothyroidism. A double-blind, placebo-controlled trial. Ann Inter Med.

[28] Nyström E, Caidahl K, Fager G (1988). A double-blind crossover 12-month study of a L-thyroxine treatment of women with “sub-clinical” hypothyroidism. Clin Endocrinol (Oxf).

[29] Jaeschke R, Guyatt G, Gerstein H (1996). Does treatment with L-thyroxine influence health status in middle-aged and older adults with subclinical hypothyroidism?. J Gen Intern Med.

[30] Kong WM, Scheikh M, Lumb P (2000). A randomized controlled trial of thyroxine treatment in mild subclinical hypothyroidism. Program & Abstracts: the Endocrine Society's 82 Annual Meeting, Toronto, June 21-24, 2000.

[31] Meier C, Roth CB, Huber G (2000). Clinical and metabolic effects of thyroxine replacement in patients with mild thyroid failure: results from a double-blind placebo-controlled study. Program & Abstracts: the Endocrine Society's 82 Annual Meeting, Toronto, June 21-24, 2000.

[32] Danese MD, Powe NR, Sawin CT (1996). Screening for mild thyroid failure at the periodic health examination: a decision and cost-effectiveness analysis. JAMA.

[33] Tanis BC, Westendorp GJ, Smelt HM (1996). Effect of thyroid substitution on hypercholesterolemia in patients with subclinical hypothyroidism: a reanalysis of intervention studies. Clin Endocrinol (Oxf).

[34] Hak AE, Pols HAP, Visser TJ (2000). Subclinical hypothyroidism is an independent risk factor for atherosclerosis and myocardial infarction in elderly women: the Rotterdam Study. Ann Intern Med.

[35] Muller B, Zulewski H, Huber P (1995). Impaired action of thyroid hormone associated with smoking in women with hypothyroidism. New Engl J Med.

[36] Stamler J, Fortmann SP, Levy RI, Prineas RJ, Tell G (1999). Primordial prevention of cardiovascular disease risk factors: panel summary. Prev Med.

[37] Bosma H, Peter R, Siegrist J, Marmot M (1998). Two alternative job stress models and the risk of coronary heart disease. Am J Public Health.

[38] Kaplan GA, Keil JE (1993). Socioeconomic factors and cardiovascular disease: a review of the literature. Circulation.

[39] Murata M, Kawano K, Matsubara Y, Ishikawa K, Watanabe K, Ikeda Y (1998). Genetic polymorphisms and risk of coronary artery disease. Semin Thromb Hemost.

[40] Bisi H, Fernandes VSO, Camargo RAC, Kock L, Abdo AH, Brito T (1989). The prevalence of unsuspected thyroid pathology in 300 sequential autopsies, with special reference to the incidental carcinoma. Cancer.

[41] Bisi H, Fernandes VSO, Camargo RYA, Longatto A, Ruggeri GB, Abdo AH (1995). Neoplastic and non-neoplastic thyroid lesions in surgical material: historical review of five decades in São Paulo, Brazil. Tumori.

[42] Knobel M, Barca MF, Pedrinola F, Medeiros G (1994). Prevalence of anti-thyroid peroxidase antibodies in autoimmune and non-autoimmune thyroid disorder in a relatively low-iodine environment. J Endocrinol Invest.

[43] Tomimori E, Pedrinola F, Cavalieri H, Knobel M, MedeirosNeto G (1995). Prevalence of incidental thyroid disease in a relatively low iodine intake area. Thyroid.

[44] Grodstein F, Stampfer MJ, Colditz GA (1997). Postmenopausal hormone therapy and mortality. N Engl J Med.

[45] Hulley S, Grady D, Bush T (1998). Randomized trial of estrogen plus progestin for secondary prevention of coronary heart disease in postmenopausal women. Heart and Estrogen Progestin Replacement Study (HERS) Research Group. JAMA.

[46] Women's Health Initiative WHI HRT Update, April, 2000 (mimeo).

[47] Hay JW, Yu WM, Ashraf T (1999). Pharmacoeconomics of lipid-lowering agents for primary and secondary prevention of coronary artery disease. Pharmacoeconomics.

[48] Hagerty JJ, Stern RA, Mason GA (2000). Subclinical hypothyroidism: a modifiable risk factor for depression?. Am J Psychiatr.

[49] Lotufo PA (1997). Non-communicable diseases in Brazil: mortality patterns, morbidity studies and risk factors. Arch Latinoam Nutr.

